# The role of anoctamin 1 in liver disease

**DOI:** 10.1111/jcmm.18320

**Published:** 2024-04-29

**Authors:** Xin Li, Yongfeng Wang, Li Zhang, Shun Yao, Qian Liu, Hai Jin, Biguang Tuo

**Affiliations:** ^1^ Department of Gastroenterology, Digestive Disease Hospital Affiliated Hospital of Zunyi Medical University Zunyi China; ^2^ The Collaborative Innovation Center of Tissue Damage Repair and Regenerative Medicine of Zunyi Medical University Zunyi China

**Keywords:** ANO1, anoctamin 1, chloride channels, HCC, liver diseases

## Abstract

Liver diseases include all types of viral hepatitis, alcoholic liver disease (ALD), nonalcoholic fatty liver disease (NAFLD), cirrhosis, liver failure (LF) and hepatocellular carcinoma (HCC). Liver disease is now one of the leading causes of disease and death worldwide, which compels us to better understand the mechanisms involved in the development of liver diseases. Anoctamin 1 (ANO1), a calcium‐activated chloride channel (CaCC), plays an important role in epithelial cell secretion, proliferation and migration. ANO1 plays a key role in transcriptional regulation as well as in many signalling pathways. It is involved in the genesis, development, progression and/or metastasis of several tumours and other diseases including liver diseases. This paper reviews the role and molecular mechanisms of ANO1 in the development of various liver diseases, aiming to provide a reference for further research on the role of ANO1 in liver diseases and to contribute to the improvement of therapeutic strategies for liver diseases by regulating ANO1.

## 1 INTRODUCTION

The liver is an important organ for maintaining normal life activities in the human body and plays an important role in blood volume regulation, immune system support, metabolism of various nutrients and chemicals, secretion of bile, synthesis and catabolism of proteins, and elimination of toxins from the body.^1^ Many drugs, environmental toxicants and dietary components may cause liver injury by inducing oxidative stress.^2^ When oxidative damage occurs in the liver, it causes a series of pathological changes, such as dysregulation of lipid metabolism in hepatocytes (hepatic steatosis), impaired liver function (hepatocyte degeneration and death) and activation of the immune response (inflammation and fibrosis/cirrhosis).^3^, ^4^ Liver diseases mainly include chronic HBV and HCV infections and autoimmune liver disease. alcoholic liver disease (ALD), nonalcoholic fatty liver disease (NAFLD), cirrhosis, liver failure (LF) and hepatocellular carcinoma (HCC).^5^ Cirrhosis and HCC are the ultimate causes of most chronic liver diseases.^6^ The global burden of both acute and chronic liver disease is enormous.^7^ From 2010 to 2019, the global burden of cirrhosis incidence increased by 12.9%.^8^ In 2020, approximately 905,700 people were diagnosed with liver cancer, and approximately 830,200 people died from liver cancer worldwide.^9^ Although the burden of liver disease is increasing, liver disease‐related mortality may be underestimated.^10^ Surveys have shown that approximately 2 million people die each year due to liver disease, accounting for 4% of all deaths.^8^, ^11^ Liver diseases have become one of the leading causes of disease and death worldwide.^12^ Therefore, it is important to clarify the mechanism of liver disease development and identify potential therapeutic targets for this disease.

Calcium‐activated chloride channels (CaCCs) are widely expressed in human tissues and organs, including neurons,^13^ secretory epithelial cells,^14^ smooth muscle cells,^15^ and cardiomyocytes,^16^ and are involved in many important physiological functions in the human body, For example, the fertilization of oocytes,^17^ transepithelial ion/fluid transport,^18^ cardiomyocyte repolarization and action potential generation,^19^ olfactory transduction^20^ and smooth muscle contraction.^15^ In 2008, three independent research groups demonstrated that anoctamin 1 (ANO1) is a member of CaCCs using different research methods.^21^, ^22^, ^23^ ANO1 is also known as transmembrane protein 16A (TMEM16A), gastrointestinal mesenchymal stromal tumour 1 (DOG1), oral cancer overexpressed 2 (ORAOV2), and tumour‐amplified and overexpressed 2 (TAOS2). As a CaCC protein, it is widely expressed in vivo.^21^, ^22^, ^23^, ^24^ Compared to that in other tissues, ANO1 is more highly expressed in the liver and gallbladder, reproductive system tissues, salivary glands, adrenal glands, and intestine^25^ (Table 1). A large body of literature suggests that ANO1 plays an important role in many pathophysiological processes, including oxidative stress,^26^ the inflammatory response,^27^ smooth muscle contraction,^15^ secretory epithelium secretion,^18^ slow‐wave activity in the gastrointestinal tract,^28^ cell proliferation^13^, ^29^ and tumour^13^, ^29^ development. Many of these pathophysiological processes are closely related to the development of liver diseases (e.g. hepatic ischemia–reperfusion injury (IRI)^30^ NAFLD,^31^ portal hypertension (PHT),^32^ and HCC^33^), which provides new insights into the pathogenesis of liver diseases.

**TABLE 1:** Expression and physiological function of Ano1 in organs and tissues.

Tissue or organ	Cellular localization	Function	Refs
Liver	Biliary epithelial cells, portal smooth muscle cells	Ion channels, cell proliferation, smooth muscle contraction	34, 87
Lungs	Alveolar epithelial cells, bronchial epithelial cells, smooth muscle cells of the airways, pulmonary microvascular endothelial cells, pulmonary fibroblasts	Ion channels, smooth muscle contraction, tracheal development	15, 132, 133, 134
Salivary gland	Salivary gland follicular cells	Ion channel	134
Thyroid gland	Thyroid follicular cells	Iodide output	135
Stomach, duodenum, colorectum	Intestinal epithelial cells, ICC	Ion channels, gastrointestinal neurotransmitter transmission, and gastrointestinal pacing activity	70, 134, 136
Pancreatic	Pancreatic alveolar cells, β‐cells	Ion channel	137
Kidney	Renal epithelial cell	Ion channels, development of renal units	138, 139
Organization of the reproductive system	Smooth muscle cells of the genital ducts, fallopian tubes and epididymal ducts	Smooth muscle contraction, which facilitates the propulsion of the egg and sperm.	69

Abbreviations: ANO1, anoctamin 1; ICC, interstitial cells of cajal.

The liver is a major site of glutathione (GSH) production and export.^34^ Studies have shown that the liver can be protected from oxidative damage by the regulation of GSH in hepatocytes.^3^ GSH is mainly synthesized in cytoplasmic lysates and transported from mitochondria, the endoplasmic reticulum, etc.,^35^, ^36^ where it further promotes oxidative metabolism, the modulation of detoxification reactions, etc., through the catalysis of glutathione‐S‐transferase (GST) and GSH peroxidase (GPx),^37^ which are also involved in the function of the liver.^1^ The transport of GSH in mitochondria is mainly regulated by membrane permeability, ATP, and GSH transporter proteins.^38^ When oxidative stress, increased Ca^2+^ influx, and GSH depletion occur, mitochondrial membrane permeability increases and GSH transport increases.^39^ This may be due to the interaction of membrane‐bound GSH transferase on the inner mitochondrial membrane with cyclophilin D, which promotes oxidant‐induced increases in membrane permeability.^40^ Interestingly, ANO1 can be expressed in mitochondrial membranes and regulates the opening of mitochondrial membranes in a procyclin D‐dependent manner,^41^ suggesting that ANO1 may play an important role in GSH transport. In addition, ANO1 acts as a CaCC, and increased intracellular Ca^2+^ activates ANO1,^42^ which is also associated with GSH transport. Similarly, GSH acts as a protective factor for the liver, ensuring the reduction of lipid peroxidation.^3^ Conversely, lipid peroxidation can activate ANO1, which can also cause lipid peroxidation.^26^, ^43^ This finding also suggested that ANO1 is associated with GSH. Furthermore, it has also been demonstrated that ROS scavengers, such as GSH and the natural parent compound coenzyme Q10, inhibit ANO1 activation.^26^ In conclusion, the relationship between ANO1 and GSH deserves further investigation. Thus, ANO1 may play an important role in liver diseases caused by impaired GSH regulation (e.g. NAFLD^31^ and cholestasis^44^). In this paper, we reviewed the role of ANO1 in the development of several liver diseases and provided strategies or directions for the diagnosis and treatment of liver diseases.

## 2 STRUCTURE, ACTIVATION AND FUNCTION OF ANO1

### 2.1 The structure of ANO1

**FIGURE 1:**
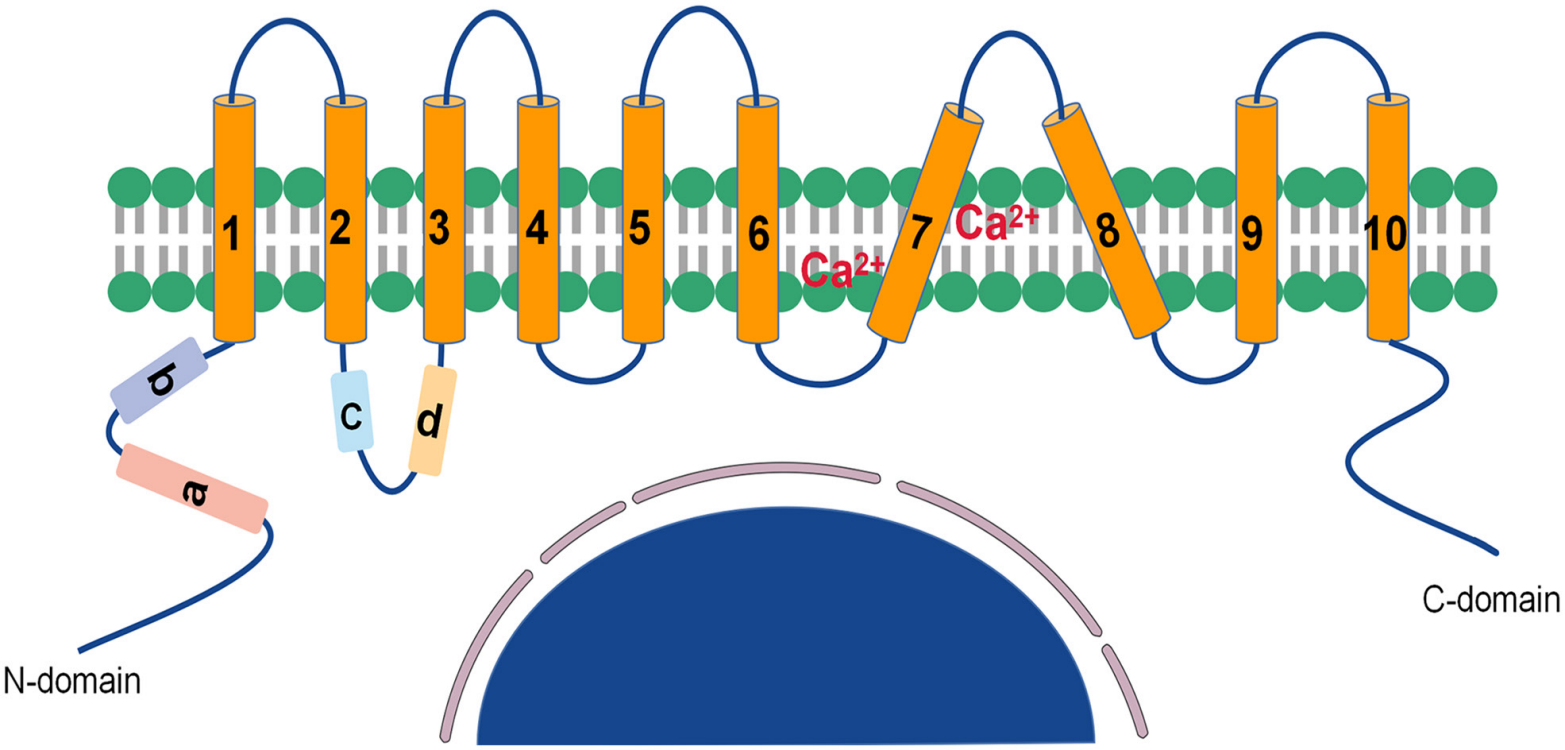
Schematic structure of ANO1.

The ANO1 protein is encoded on human chromosome 11q13.^18^ ANO1 has a molecular weight of 114 kDa,^23^ consists of 960 amino acids,^23^ and is expressed predominantly at the plasma membrane.^45^ In 2014, Brunner and colleagues^46^ identified the X‐ray crystal structure of an ANO1 homologue and an ANO1 homologue (nhANO1) from the Nectria haematococca that functions as a Ca^2+^‐activated phospholipid scramblase. The overall structure of mouse ANO1 was found to be similar to that of nhANO1, which is composed of two identical subunits.^47^ Each subunit contains two Ca^2+^‐binding sites and 10 transmembrane structural domains (TMs),^46^, ^47^ and each subunit has cytoplasmic N and C termini^46^ (Figure 1). Both termini are exposed on the cytoplasmic side of the membrane.^46^ ANO1 contains two ion‐conducting pores, each of which is activated mostly independently.^47^ Recent studies have identified two Ca^2+^‐binding sites within the ion‐conducting pore,^48^, ^49^ where Ca^2+^ binding triggers a conformational change in the α‐helix that results in a conduction function.^47^, ^50^ The ANO1 mRNA sequence is alternatively spliced and can generate protein isoforms with selective protein fragments a (116 amino acids), b (22 amino acids), c (4 amino acids) and d (26 amino acids) in various combinations.^21^, ^51^ Fragments a and b are located in the N‐terminus and fragments c and d are located in the first intracellular loop. Fragments b and c may be part of the protein region involved in voltage and Ca^2+^ sensing, and deletion of fragment b increases Ca^2+^ sensitivity, whereas deletion of fragment c decreases the apparent Ca^2+^ sensitivity and increases the voltage‐dependent activity of ANO1 channels.^51^, ^52^

### 2.2 Activation of ANO1

ANO1, as a CaCC, is mainly regulated by the intracellular Ca^2+^ concentration and membrane voltage and by anion selectivity outside the cell.^42^, ^53^ Terashima et al^42^ tested the function of ANO1 in vitro. ANO1 was inactive when the membrane voltage or the Ca^2+^ concentration was 0, ANO1 was activated when the membrane voltage was increased or when the Ca^2+^ concentration increased and ANO1 was activated; when the membrane voltage V ∼ +140 mV or saturated Ca^2+^ was added, it caused strong exocytosis of Cl^−^ and the exocytosis of Cl^−^ was maximal when the two acted synergistically.^42^ Interestingly, in the oligomeric state, the activation of ANO1 was not affected by changes in intracellular Ca^2+^.^54^ ANO1 exists in multiple open states and has different ionic selectivities.^49^ The order of anionic selectivity of ANO1 is NO_3_^−^ > I^−^ > Br^−^ > Cl^−^ > F^−^.^22^, ^23^, ^55^ Replacing arginine and lysine residues with glutamate in the hypothetical pore loop between transmembrane fragments 5 and 6 has also been reported to alter ion selectivity.^23^ In addition, molecules such as calmodulin,^56^ high extracellular concentrations of protons,^57^ cholesterol, and phosphatidylinositol can activate ANO1, furthering its role.^58^ ANO1 is regulated by many molecules, such as calmodulin, protons, cholesterol and phosphatidylinositol, as well as by various stimuli, such as thermal and mechanical stimuli.^58^ Interestingly, calmodulin is not needed for ANO1 to function, as Ca^2+^ can directly bind to ANO1 and induce its function.^42^ Whether calmodulin mediates the Ca^2+^‐dependent activation of ANO1 is still controversial. Furthermore, it has been demonstrated that ANO1 can be directly activated when the temperature exceeds 44°C.^59^ Although ANO1 is regulated by many molecules, the regulatory effects of other molecules on ANO1 need to be further explored in the future.

### 2.3 Functions of ANO1

To date, ANO1 has been found to be involved in the physiopathological processes of a variety of organs and tissues in the human brain,^13^ heart,^16^ airway,^14^ urinary system,^15^ gastrointestinal tract,^60^ liver,^33^ and so on. It is well known that ANO1 plays important roles in transepithelial transport,^18^ smooth muscle contraction,^55^ and signalling.^61^ Previous studies have also demonstrated that ANO1 is involved in the proliferation of mesenchymal stromal cells in Cajal, which is important for slow‐wave activity in the gastrointestinal tract.^28^ ANO1 has also been suggested to be required for normal tracheal development in mice.^62^ Prior to the maturation of the auditory system, the activation of ANO1 channels on supporting cells in the cochlea promotes the maturation of the hearing system by depolarizing hair cells and spiral ganglion neurons in the cochlea and generating self‐generating activity.^63^ Interestingly, ANO1 is also involved in the maturation of glial cells in the brain and contributes to cortical development.^64^ Additionally, ANO1 in cholinergic neurons has been associated with anxiety‐related behaviours.^65^ In addition, ANO1 improves vascular remodelling by inhibiting autophagy and further inhibiting the proliferation of vascular smooth muscle cells.^66^ Additionally, ANO1 has been associated with improved endothelial function,^67^ blood pressure regulation, and ventricular hypertrophy.^68^

Interestingly, ANO1 not only acts as a heat sensor and thus mediates acute thermal hyperalgesia but also has the ability to increase the excitability of dorsal root ganglion neurons under inflammatory or neurogenic conditions, thereby exacerbating the degree of pathological pain caused by inflammation or tissue damage.^69^ In addition, ANO1 promotes proliferation,^70^ migration and invasion in certain cancers.^71^ Additionally, ANO1 has been associated with apoptosis in a variety of cancers.^72^, ^73^ In addition, amplification of the ANO1 locus has been associated with poor prognosis, suggesting that it may be a potential prognostic marker.^74^, ^75^ The functions of ANO1 are gradually being elucidated, but many functions remain undefined and need to be further explored in the future.

## 3 PHYSIOLOGICAL ROLE OF ANO1 IN THE LIVER

### 3.1 ANO1 and bile secretion

Bile consists of water, bile pigments, cholesterol, lecithin, sodium (Na^+^), potassium (K^+^), calcium (Ca^2+^) and bicarbonate (HCO_3_^−^).^76^ Bile originates from hepatocytes and matures through the regulation of bile duct epithelial uptake and secretion by the transport system.^77^ Purinergic signalling (P2) transduction is an important pathway for cholangiocyte transport and bile formation.^78^ Cl^−^ channels in the parietal membrane of bile duct cells provide the driving force for bile secretion.^79^ ANO1 is expressed in human mouse and rat biliary epithelial cells (BECs).^80^ ANO1 is one of the key members of the purinergic signalling pathway.^81^ ATP from bile binds to P2 receptors on bile duct cells, a process that activates ANO1, thereby increasing ductal Cl^−^ transport.^81^ Bile acids can activate membrane ANO1 during extracellular ATP and Ca^2+^ I regulation, thereby stimulating Cl^−^ secretion in mouse and human cholangiocytes.^82^ Increased fluid flow or increased shear stress located on the apical membrane of BECs can potently stimulate ATP release.^83^ An increase in bile viscosity can also stimulate ATP release, and the released ATP binds to autocrine/paracrine P2 receptors, leading to an increase in intracellular Ca^2+^ and activation of ANO1, causing an ANO1‐mediated increase in membrane Cl^−^ permeability and thus bile dilution.^80^ In BECs, ATP in bile induces protein kinase C (PKC) to move from the cytoplasm to the plasma membrane, activating ANO1 and stimulating Cl^−^ secretion.^84^ An increase in luminal Cl^−^ can stimulate Cl^−^/HCO_3_^−^ exchange via an anion exchanger (AE) and stimulate the opening of water channels, leading to bile secretion and dilution.^85^ The above results suggest that ANO1 is involved in bile secretion and bile dilution. In addition, GSH translocation into bile is also a driver of bile secretion.^86^ Previous studies have shown that GSH inhibits ANO1 activation,^26^ suggesting that both GSH and ANO1 play a role in bile secretion. It has been shown that oxidative stress can induce cholestasis and that GSH, an oxygen scavenger, can reduce oxidative stress.^44^ ANO1 can also cause oxidative stress and exacerbate oxidative damage in the liver. Thus, regulating ANO1 could provide a new therapeutic strategy for liver diseases characterized by impaired bile flow (e.g. cholestasis).

Interestingly, in intrahepatic cholangiocyte‐like organs (ICOs), hypoxia attenuates ANO1 activity, which reduces HCO_3_^−^/Cl^−^ exchange, decreases bicarbonate secretion in ICOs and impairs cholangiocyte resistance to the cytotoxic effects of bile.^87^ Interleukin‐4 (IL‐4) and interleukin‐13 (IL‐13) increase ANO1 expression in the liver through specific Janus kinase (JAK)/activator of transcription (STAT) signalling, which leads to an increase in Cl^−^ currents and promotes biliary epithelial Cl^−^ secretion in the biliary epithelium.^88^ ANO1 is also present in Cajal interstitial cells (ICCs) of the gallbladder, where it participates in muscle contraction.^89^ When ANO1 is inhibited, muscle contraction slows.^90^ A decrease in the number of ICCs in the gallbladder slows gallbladder peristalsis and promotes gallstone production.^91^ We propose that ANO1 in the gallbladder reduces gallstone production by promoting gallbladder peristalsis, but the exact mechanism involved needs to be better elucidated. It might also be investigated whether ANO1 plays an important role in liver diseases characterized by impaired bile flow.

### 3.2 ANO1 and portal blood pressure regulation

The portal vein is the blood vessel of the liver connecting the gastrointestinal tract and the spleen (internal organs) and is the main source of blood for the liver.^92^ ANO1 is present in portal vein smooth muscle cells (PVSMCs).^18^ Zeng et al^32^ reported that ANO1 overexpression promoted PVSMC proliferation and that the inhibition of ANO1 suppressed this process in vitro, suggesting that ANO1 is involved in the proliferation of PVSMCs. Flow cytometry studies showed that ANO1 promoted cell proliferation and vascular remodelling by promoting the entry of cells in the G0/G1 phase into the S phase. Previous studies have also shown that ANO1 is involved in the regulation of vascular pressure.^93^ Interestingly, in vivo experiments yielded opposite results, with ANO1 being significantly downregulated in the portal vein of PHT rats, which may be related to the involvement of other mechanisms in the regulation of PHT in vivo.^32^ Interestingly, in vivo experiments yielded opposite results, with ANO1 expression significantly downregulated in the portal vein of rats with PHT.^32^ Notably, elevated portal pressure is the main feature of PHT,^94^, ^95^ and these findings provide new ideas for the development of PHT. In addition, a recent study showed that in PVSMCs in a model of cirrhotic PHT, angiotensin II (ANGII) reduced vascular tone and contraction by downregulating the expression of ANO1,^96^ but the underlying mechanism is unclear and requires further investigation. In addition, it has been shown that in vascular smooth muscle cells, ANO1 can improve vascular remodelling by inhibiting the formation of Bcl‐2‐p62 complexes, thereby inhibiting autophagy.^66^ In summary, ANO1 plays an important role in vascular pressure regulation and vascular remodelling in PHT and may be a potential therapeutic target.

## 4 PATHOLOGIC ROLE OF ANO1 IN THE LIVER

### 4.1 ANO1 and hepatic IRI

When blood flow is restored in transiently ischemic liver tissues, it may lead to reinjury of already ischemic liver tissues, a phenomenon known as hepatic IRI.^97^ IRI is the leading cause of LF.^98^ The inflammatory response is considered a key mechanism in the process of IRI.^30^, ^99^ By comparing ANO1 expression in the livers of humans and mice before and after partial hepatectomy, Guo et al. found that ANO1 expression was increased in hepatocytes after IRI. Further experiments revealed that the lack of hepatocyte‐specific ANO1 ameliorated inflammation in hepatic IRI, and mice overexpressing ANO1 exhibited the opposite phenotype. This finding suggested that ANO1 could be involved in promoting hepatic IRI and that IRI could also stimulate ANO1 expression, but the specific mechanism of action is not known. Ferroptosis is an iron‐dependent cell death process associated with intracellular iron accumulation, lipid peroxidation, and reactive oxygen species production.^100^ ANO1 contributes to the production of excess reactive oxygen species, which induces lipid peroxidation and leads to ferroptosis.^30^ ANO1‐mediated iron death is an upstream regulator of inflammation during hepatic IRI, and ferroptosis can promote IRI.^30^ ANO1 can promote IRI through ferroptosis. In addition, another study showed that GSH peroxidase 4 (GPX4) is a negative regulator of iron death.^101^ Overexpression of ANO1 reduces hepatic GPX4 expression, while knockdown of ANO1 seems to have the opposite effect.^30^ Guo et al.^30^ reported that ANO1 interacts with GPX4 to induce GPX4 ubiquitination and degradation, which upregulates ferroptosis. Conversely, disruption of the ANO1‐GPX4 interaction abolishes the effects of ANO1 on GPX4 ubiquitination, ferroptosis, and hepatic IRI. These results suggest that ANO1 exacerbates hepatic IRI by promoting GPX4‐dependent ferroptosis. miR‐9 downregulation promotes the inflammatory response and oxidative stress injury, promotes hepatocyte apoptosis, and exacerbates IRI; conversely, miR‐9 upregulation ameliorates IRI I.^102^ A previous study revealed that miR‐9 upregulation inhibited ANO1 expression.^103^ This finding suggested that upregulated miR‐9 may improve IRI by inhibiting ANO1, but the underlying mechanism needs to be elucidated. Therefore, the development of therapeutic agents targeting ANO1 may be a promising approach for preventing or treating hepatic IRI. ANO1 also plays a role in IRI in other organs (e.g. the lungs and kidneys), but the exact mechanism is unknown.

### 4.2 ANO1 and NAFLD

**FIGURE 2:**
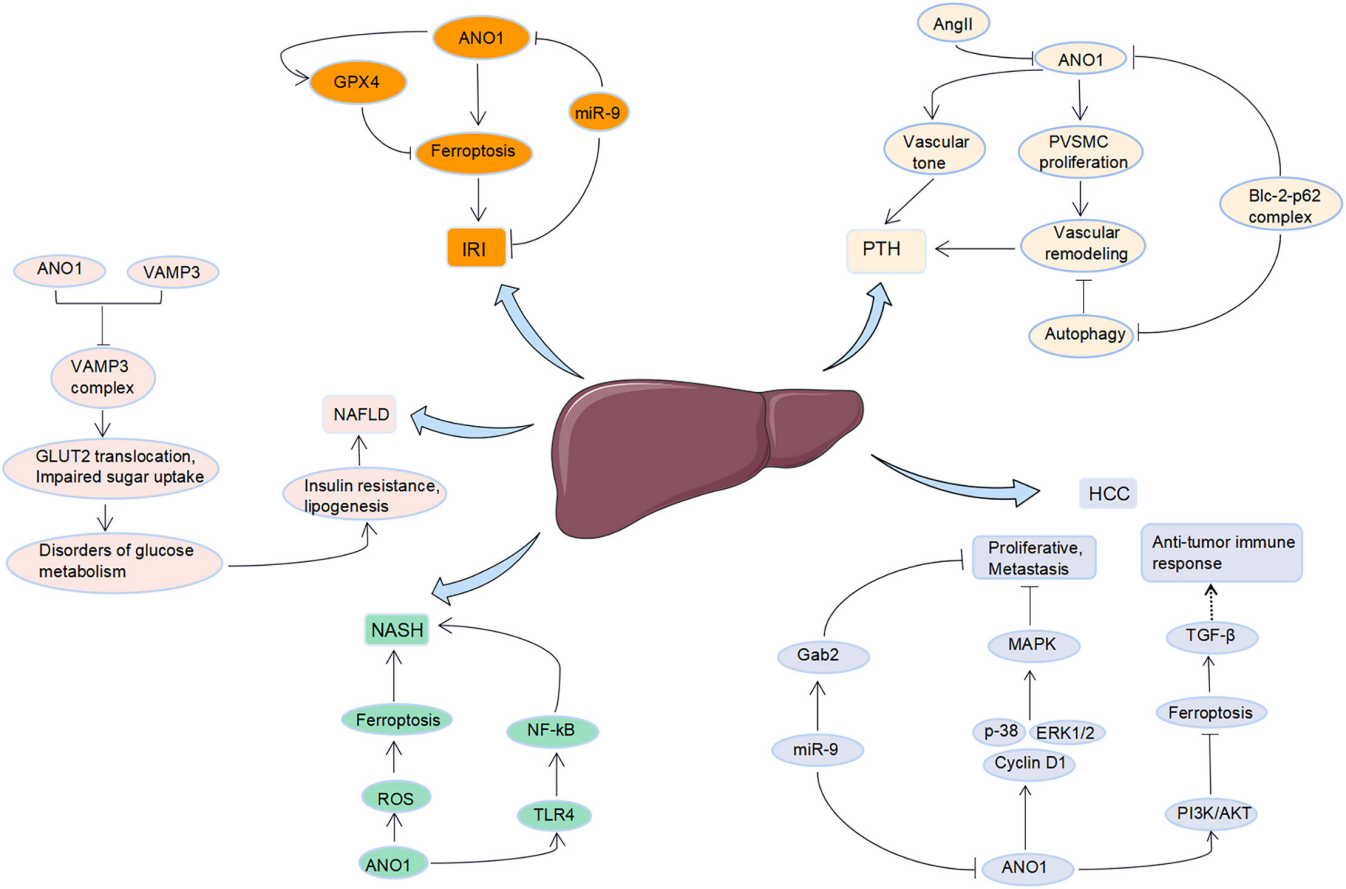
ANO1 is involved in the pathogenesis of liver disease.

NAFLD, also known as metabolic steatohepatitis, includes nonalcoholic steatohepatitis (NASH) and nonalcoholic fatty liver (NAFL).^104^ It is diagnosed in approximately 30% of the global population, has become the most common chronic liver disease in the world, and is characterized by the gradual accumulation of lipids in the liver, which usually evolves from steatohepatitis to fibrosis, cirrhosis, and even HCC.^105^ In recent years, NAFLD has received increasing attention. Guo et al^31^ reported increased expression of ANO1 in the liver of mice and patients with hepatic steatosis. As the NAFLD score increased, the abundance of ANO1 in the liver also increased, suggesting that ANO1 may play a role in hepatic steatosis. In mice with hepatocyte‐specific ANO1 overexpression, obesity and insulin resistance induced by a high‐fat diet (HFD) were markedly increased, whereas the hepatic steatosis, adipogenesis, and inflammation induced by a HFD were reduced when ANO1 was specifically knocked down. Further analysis revealed that ANO1 in hepatocytes binds to vesicle‐associated membrane protein 3 (VAMP3), leading to degradation of VAMP3, as well as inhibition of the formation of VAMP3/synuclein 4 and VAMP3/synaptosome‐associated protein 23 complexes, which results in hepatic glucose transport protein 2 (GLUT2) translocation and impaired glucose uptake, leading to disorders of glucose metabolism with the secondary effects of increased insulin resistance and promotion of adiposity.^31^ Therefore, the knockdown of ANO1 can ameliorate obesity, hepatic glucose metabolism disorders, and steatosis caused by a high‐fat diet, and ANO1 may be a potential therapeutic target for NAFLD (Figure 2).

NASH is a necroinflammatory response in which the abnormal accumulation of lipids in hepatocytes and oxidative stress induced by iron accumulation are the main triggers.^106^ Toll‐like receptor 4 (TLR4) induces inflammatory responses in adipocytes and macrophages.^107^ TLR4 expression is increased in NASH patients, and TLR4 induces increased NF‐κB nuclear translocation and activation in hepatocytes, leading to a range of inflammatory responses.^108^ ANO1 can participate in inflammatory responses through the NF‐κB signalling pathway,^27^ and ANO1 overexpression enhances the activation of the TLR4–NF‐κB signalling pathway, which enhances the inflammatory response in the liver, especially in NASH.^27^ ANO1 is linked to ferroptosis through a complex mechanism whereby ANO1 contributes to the generation of excess reactive oxygen species, thereby inducing lipid peroxidation, which further leads to ferroptosis; however, lipid peroxidation can also activate ANO1.^26^, ^43^ Another study showed that ferroptosis is an important trigger of NASH‐induced inflammation.^109^ ANO1 can promote the development of NASH through ferroptosis, but the specific mechanism involved needs to be defined. However, further experiments are needed in the future to prove this hypothesis. In addition, when ROS are elevated or GSH is depleted, a decrease in GSH content can also cause hepatocyte death and further liver inflammation.^39^ ANO1 can promote liver inflammation.^30^ However, GSH can inhibit the activation of ANO1.^26^ This finding also provides evidence that ANO1 may be a potential therapeutic target for NAFLD.

### 4.3 ANO1 and HCC

Globally, approximately 70,000 people die from HCC every year, making HCC one of the leading causes of cancer‐related death.^110^ Finding effective treatments for HCC has become a common goal worldwide.^111^ Deng et al^33^ reported that ANO1 was more highly expressed in HCC cells than in paraneoplastic tissues, and flow cytometry revealed that the proportion of SMMC‐7721 cells in the G0/G1 phase was increased, the proportion of SMMC‐7721 cells in the S phase was significantly reduced, and the cell cycle was significantly inhibited, suppressing cell proliferation but not apoptosis, suggesting that ANO1 can promote the growth of HCC cells. The same study also revealed that the knockdown of ANO1 significantly decreased p‐p38 and phosphorylation extracellular signal‐regulated kinases 1 and 2 (p‐ERK1/2) and that the cell cycle protein D1 was reduced, but p‐JNK was not altered, which suggests that in HCC cells, the knockdown of ANO1 can regulate the cell cycle and inhibit cell proliferation and metastasis through the Mitogen‐activated protein kinase (MAPK) signalling pathway. In addition, through a tumorigenicity assay in nude mice, the authors showed that the knockdown of ANO1 inhibited tumorigenicity in vivo^33^;therefore, an inhibitor of ANO1 might be helpful for the treatment of HCC. Cell cycle protein‐dependent kinase 1 (CDK1) is highly expressed in HCC tissues and is associated with poor prognosis.^112^ CDK1‐based ANO1 prognostic modelling can accurately predict the prognosis of HCC patients.^113^ ANO1 is closely associated with vascular infiltration and negatively correlated with HCC prognosis.^114^ Diltiazem is a calcium channel blocker that suppresses tumour growth by inhibiting calcium influx.^115^ Diltiazem can downregulate ANO1 at the mRNA and protein levels, thus inhibiting the proliferation and invasion of HCC cells.^116^ This finding suggested that diltiazem might play a role in the treatment of HCC, but further experimental studies are needed. miR‐9 can target the signalling integrin Gab2 to inhibit the proliferation and migration of HCC cells.^117^ miR‐9 upregulation can inhibit ANO1 expression,^103^ but whether miR‐9 further inhibits cell proliferation and migration in HCC through ANO1 still needs further investigation. Ferroptosis is a key cell death response triggered upon treatment of multiple tumours.^118^ In gastrointestinal tumours, ANO1 inhibits ferroptosis by activating phosphatidylinositol 3‐kinase (PI3K)/protein kinase B (PKB/AKT) signalling, promotes TGF‐β release, facilitates cancer progression, and promotes cancer‐associated fibroblast recruitment, which weakens CD8+ T‐cell‐mediated antitumor immune responses and generates resistance to immunotherapy.^119^ Whether ANO1 has an immunologic role in HCC is unclear. In summary, ANO1 is highly expressed in HCC and can promote the growth and progression of HCC by promoting MAPK signalling and thus the growth and progression of HCC. ANO1 may also be involved in antitumor immunity by inhibiting iron death. This evidence suggests that ANO1 may be a potential novel therapeutic target for HCC, but many studies are needed to explore this further.

### 4.4 ANO1 and other liver‐related diseases

ANO1 is also involved in other liver‐related diseases. The expression of mammalian target of rapamycin (mTOR), a serine/threonine kinase, which promotes the survival and proliferation of biliary tract cancer cells, is frequently upregulated in advanced biliary tract cancers.^120^, ^121^ Kulkarni et al^122^ reported that ANO1 can interact with the mTOR pathway to regulate the cytoskeleton, survival, proliferation, and migration in cholangiocellular carcinoma, but the specific underlying mechanisms need to be further investigated. The liver is a common site for secondary metastasis of many malignant tumours, including lung cancer, breast cancer, ESCC, gastric cancer, pancreatic cancer, CRC and prostate cancer.^123^, ^124^, ^125^ ANO1 is highly expressed in CRC, especially in liver metastases.^126^ To explore the role of ANO1 in CRC liver metastasis, Wu et al.^127^ found that when bioluminescence was imaged in the livers of mice treated with an anti‐ANO1 antibody by establishing an anti‐ANO1 antibody‐treated mouse model of CRC liver metastasis, only a few tumour nodules were observed by the naked eye when low concentrations of the anti‐ANO1 antibody were used, and virtually no tumour nodules were observed when high concentrations were used, while both concentrations reduced the serum ALT, AST and total bilirubin.

## 5 MECHANISM OF ACTION OF ANO1 IN THE LIVER

### 5.1 ANO1 functions as a chloride channel

As a CaCC, ANO1 can exert its function through chloride transport. In human and mouse cholangiocytes, when fluid flow or shear is increased, ATP is released at the parietal membrane of BECs, which increases the binding of ATP to the purinergic P2 receptor and causes ANO1 activation.^81^ At the same time, the released ATP also increases the intracellular Ca2+ concentration, activating ANO1.^82^ When ANO1 is activated, Cl^−^ is released from the cell, promoting bile secretion.^82^ In addition, when the viscosity of bile increases, it also activates ANO1, causing Cl^−^ release and diluting bile.^80^ It was also found that ANO1 acts as a chloride channel and is associated with cell proliferation. Proliferation requires the regulation of cell volume, which is key to the progression of the cell cycle from the G2 phase to the M phase.^128^ Ion channel activity has been shown to correlate with the progression of the G1/S and G2/M leaps.^128^ Currently, Cl^−^ channels are thought to be associated with the regulation of cell volume and cell proliferation.^129^ Mechanistic analyses have shown that in PVSMCs, ANO1 promotes cell proliferation by facilitating cell progression from the G0/G1 phase to the S phase.^32^ In contrast, in SMMC‐7721 cells (HCC cells) in which ANO1 was knocked down, the ratio of cells in the G0/G1 phase was increased, and the proportion of cells in the S phase was significantly decreased, which caused cell cycle arrest and thus inhibited cell proliferation.^33^ Interestingly, a reduction in the intracellular Cl^−^ concentration also promotes NF‐κB signalling activation.^130^ It is evident that ANO1 activation also activates NF‐κB signalling by reducing the intracellular Cl^−^ concentration, which is involved in hepatocyte inflammation.

### 5.2 ANO1 interacts with other proteins

Several studies have shown that ANO1 can also be involved in the pathogenesis of liver diseases by interacting with other proteins. In hepatic IRI, ANO1 interacts with GPX4, which can induce GPX4 ubiquitination and degradation, thereby enhancing iron death and causing IRI.^30^ Conversely, disruption of the ANO1‐GPX4 interaction eliminates the effects of ANO1 on GPX4 ubiquitination, iron death and hepatic IRI.^30^ In NAFLD, ANO1 binds to vesicle‐associated membrane protein 3 (VAMP3), leading to degradation of VAMP3, as well as inhibition of the formation of VAMP3/synuclein 4 and VAMP3/synaptosome‐associated protein 23 complexes, which results in hepatic glucose transport protein 2 (GLUT2) translocation and impaired glucose uptake, causing disruption of glucose metabolism with secondary effects, increasing insulin resistance and promoting adipogenesis.^31^ In addition, Kulkarni et al^122^ reported that ANO1 can interact with mTOR to regulate the cytoskeleton, survival, proliferation and migration in cholangiocarcinoma.

In addition, ANO1 is involved in the regulation of multiple liver disease signalling pathways. These pathways include the MAPK signalling pathway,^33^ Toll‐like receptor 4 (TLR4)/nuclear factor κ‐light chain‐activated B‐cell enhancer (NF‐κB) signalling,^31^ and JAK/STAT signalling.^84^ Knockdown of ANO1 in HCC cells resulted in a significant decrease in p‐p38 and p‐ERK1/2, a decrease in the cell cycle protein D1, and inhibition of HCC cell proliferation, migration and invasion.^33^ ANO1 can regulate the proliferation, migration and invasion of HCC cells through the MAPK signalling pathway.^33^ The Th2 cytokines interleukin‐4 (IL‐4) and interleukin‐13 (IL‐13) increase the gene transcription of ANO1 through JAK transcriptional activator of transcription 6 (STAT6) signalling to upregulate ANO1 expression in BECs and cholangiocytes, which is further involved in bile secretion.^88^ Previous studies have shown that ANO1 can participate in inflammatory responses through the NF‐κB signalling pathway.^27^ In addition, in NASH, ANO1 overexpression enhances the activation of the TLR4‐NF‐κB signalling pathway, which enhances the inflammatory response in the liver.^108^

## 6 CONCLUSIONS

There is growing evidence that ANO1 is a CaCC that is involved in a variety of pathophysiological processes involving chloride ions, including pain,^131^ the inflammatory response,^27^ smooth muscle contraction,^15^ secretory epithelium secretion,^18^ slow‐wave activity in the gastrointestinal tract,^28^ cell proliferation^13^, ^29^ and tumour^13^, ^29^ development and that ANO1 is likewise important in liver disease development. In BECs, ATP binding to the P2 receptor and increased Ca^2+^ ^82^ can activate ANO1,^81^ and JAK/STAT signalling increases ANO1 expression in the liver, which increases ductal Cl^−^ transport^88^ and participates in the regulation of bile production and dilution. It has been shown that ANO1 induces ferroptosis through a variety of complex mechanisms and that ferroptosis is associated with intracellular iron accumulation, lipid peroxidation, and reactive oxygen species production.^30^ Ferroptosis is widely involved in liver diseases, including IRI, NASH, and HCC. ANO1 can exacerbate hepatic IRI by promoting GPX4‐dependent iron death,^30^ and ferroptosis is also an important trigger of inflammation triggered by NASH.^109^ However, whether iron‐mediated death plays an antitumor immune role in HCC^119^ needs to be further investigated in the future. In addition, ANO1 promotes hepatic steatosis, induces glucose metabolism disorders, and facilitates the development of NAFLD.^31^ In PVSMCs, high expression of ANO1 promotes the proliferation of PVSMCs and participates in the regulation of PHT.^32^ In addition, ANO1 is highly expressed in HCC, and when it is knocked down, ANO1 regulates the cell cycle and inhibits cell proliferation and metastasis through the MAPK signalling pathway^33^; however, the mechanism of action of ANO1 overexpression in HCC is still unclear and needs to be further investigated in the future. ANO1 is also involved in secondary liver metastasis of CRC^127^ and may be a potential therapeutic target. However, whether ANO1 is also involved in other secondary metastases of tumours needs further study. The above evidence suggests that aberrant expression and activation of ANO1 in ANO1 play important roles in liver disease, which will aid in the development of drug therapies targeting ANO1. The specific mechanisms of certain pathologies involving ANO1 in the liver have not been elucidated and warrant in‐depth exploration.

## AUTHOR CONTRIBUTIONS

**Xin Li:** Writing – original draft (lead). **Yongfeng Wang:** Investigation (equal). **Li Zhang:** Resources (supporting). **Shun Yao:** Resources (supporting). **Qian Liu:** Resources (supporting). **Hai Jin:** Funding acquisition (supporting); project administration (supporting); resources (supporting); supervision (supporting); writing – review and editing (supporting). **Biguang Tuo:** Funding acquisition (supporting); writing – review and editing (supporting).

## FUNDING INFORMATION

The present study was supported by grants from the National Natural Science Foundation of China (grant nos. 81960507, 82073087 and 82160112), the Collaborative Innovation Center of the Chinese Ministry of Education (2020–39), the Science and Technology Bureau fund of Zunyi City [grant no. ZUN SHI KE HE HZ ZI (2019) 93‐Hao] and the Science and Technology Plan Project of Guizhou Province [grant nos. QIAN KE HE JI CHU‐ZK (2021) YI BAN451 and QIAN KE HE LH ZI (2017) 7095 HAO].

## CONFLICT OF INTEREST STATEMENT

The authors declare that they have no competing interests.

## Data Availability

Not applicable.
